# Supraspinal facilitation of painful stimuli by glutamatergic innervation from the retrosplenial to the anterior cingulate cortex

**DOI:** 10.1371/journal.pbio.3003011

**Published:** 2025-01-27

**Authors:** Shun Hao, Man Xue, Qi-Yu Chen, Jinjin Wan, Yu-Jie Ma, Wantong Shi, Xuanying Chen, Xu-Hui Li, Jing-Shan Lu, Fang Xu, Guo-Qiang Bi, Wucheng Tao, Min Zhuo

**Affiliations:** 1 Key Laboratory of Brain Aging and Neurodegenerative Diseases, Fujian Medical University, Fuzhou, China; 2 Oujiang Laboratory, Zhejiang Lab for Regenerative Medicine, Vision and Brain Health, Wenzhou, Zhejiang, China; 3 Zhuomin Institute of Brain Research, Forevercheer Medicine Pharmac Inc., Qingdao, Shandong, China; 4 Department of Pharmacy, Shenzhen Hospital, Southern Medical University, Shenzhen, China; 5 CAS Key Laboratory of Brain Connectome and Manipulation, Interdisciplinary Center for Brain Information, The Brain Cognition and Brain Disease Institute, Shenzhen Institute of Advanced Technology, Chinese Academy of Sciences, Shenzhen, Guangdong, China; 6 Institute of Neuroscience, Kunming Medical University, Kunming, Yunnan, China; 7 Center for Neuron and Disease, Frontier Institute of Science and Technology, Xi’an Jiaotong University, Xi’an, Shaanxi, China; 8 Department of Physiology, Faculty of Medicine, University of Toronto, Medical Science Building, 1 King’s College Circle, Toronto, Ontario, Canada; Columbia University Irving Medical Center, UNITED STATES OF AMERICA

## Abstract

The anterior cingulate cortex (ACC) is recognized as a pivotal cortical region involved in the perception of pain. The retrosplenial cortex (RSC), located posterior to the ACC, is known to play a significant role in navigation and memory processes. Although the projections from the RSC to the ACC have been found, the specifics of the synaptic connections and the functional implications of the RSC-ACC projections remain less understood. In this study, we employed a combination of whole-brain imaging, in vitro electrophysiology, and two-photon calcium imaging techniques to confirm the presence of direct excitatory glutamatergic projections from the RSC to the ACC in mice. This excitatory transmission is predominantly mediated by the postsynaptic AMPA receptors. Furthermore, the activation of the RSC-ACC projections through opto-/chemogenetics significantly facilitated the behavioral responses to both mechanical and thermal nociceptive stimuli in adult mice. Notably, this activation did not influence spinal nociceptive responses in the tail-flick test, nor did it affect anxiety-like or aversive behaviors. These findings indicate that the RSC-ACC glutamatergic pathway modulates nociceptive perception primarily at the supraspinal cortical level. We have identified a novel cortico-cortical facilitatory pathway that contributes to nociceptive processing in the cingulate cortex. The RSC-ACC pathway probably serves to integrate memory engrams with pain perception in both humans and animals.

## Abstract 2

Most studies on nociceptive pathways focus on links between the subcortical regions and spinal cord, but do cortico-cortical circuits also contribute to pain modulation? This study reveals a pathway from the retrosplenial cortex to the anterior cingulate cortex that modulates nociceptive perception.

## Introduction

The anterior cingulate cortex (ACC) is recognized for its important roles in pain perception, emotional, and cognitive regulation [1–3]. As a high cortex of pain processing, the ACC integrates sensory, nociceptive, and emotional information from distinct brain regions, including the thalamus, amygdala, prefrontal cortex, somatosensory cortex, and so on [1,3–8]. In human studies, both acute and chronic pain stimuli have been shown to induce transient and persistent hyperactivity respectively in the ACC [9–11]. Additionally, the activation of the ACC also mediates the emotional pain in both humans and animals [3]. Brain-imaging studies in humans proved that induced sadness and social exclusion or rejection elevated the activity of the ACC [12,13]. The studies in freely moving animals showed that pharmacological or electrical activation of the ACC could induce emotional fear and aversive response [14–16]. Pharmacological inhibition of ACC activity or synaptic plasticity alleviated pain and pain-related unpleasant responses in animals [17–19]. These findings consistently indicate that the ACC is crucial for pain perception and related unpleasant feelings.

In recent years, accumulative studies focused on the roles of the communications between the ACC and other brain regions in pain and pain-related emotions. The ACC participated in more complicated pain modulation through broad anatomic connections with different brain regions [20,21]. For example, a recent study showed that the projections from the ACC to the nucleus accumbens (NAc) mediated the empathy pain, including the social transfer of both pain and analgesia [20]. The activation of oxytocin-projecting fibers from the paraventricular nucleus (PVN) to ACC attenuated neuropathic pain and pain-induced anxiety behaviors [22]. A positive-feedback loop between the ACC and ventral tegmental area (VTA) has been reported to mediate the progression and maintenance of persistent pain and comorbid anxiodepressive-like behavior [23]. Besides the subcortical regions, an increasing number of cortico-cortical connections related to the ACC are found to be involved in the processing of nociceptive and emotional information. The inputs from the primary somatosensory cortex (S1) to the ACC contributed to chronic pain by modulating neuronal activity in the ACC [8]. Another study showed that activation of the S1 axon terminals enhanced the response of ACC neurons to nociceptive stimuli, causing pain-aversive behaviors in rats [5]. The GABAergic neurons in the ACC also received the glutamatergic projections from the medial part of the secondary visual cortex (V2M). Low-intensity green light activated glutamatergic projections from V2M^Glu^ to ACC^GABA^. This inhibited the local glutamatergic neurons in the ACC, causing green-light-induced antinociceptive effects [24]. Obviously, there remains great potential for exploring the functions of cortico-cortical pathways associated with the ACC in the modulation of pain and related emotions according to its complicated neural connections.

The retrosplenial cortex (RSC), located posterior to the ACC, exerts many high cognitive functions, including spatial navigation, episodic memory, and imagination [25]. It is recognized that the RSC neurons are responsible for encoding long-term memories related to sensory, motional, and spatial information [26]. Previous imaging studies suggested that the activity of the RSC was associated with chronic pain conditions [27,28]. However, the direct and further evidence remains insufficient. In this study, we employed a novel microscopy technique called Volumetric Imaging with Synchronized on-the-fly-scan and Readout (VISoR) to label all the afferent neurons to the ACC in adult mice. The anatomical results showed plenty of projections from the RSC to ACC. Furthermore, we utilized viral tracing, electrophysiology, opto-/chemogenetics, and two-photon calcium imaging to explore the characteristics of the RSC-ACC projections in pain processing. Our results revealed a new cortico-cortical glutamatergic pathway for specifical facilitation of mechanical and thermal nociceptive perception at a supraspinal level.

## Results

### The substantial projections from the RSC to the ACC

**Fig 1:**
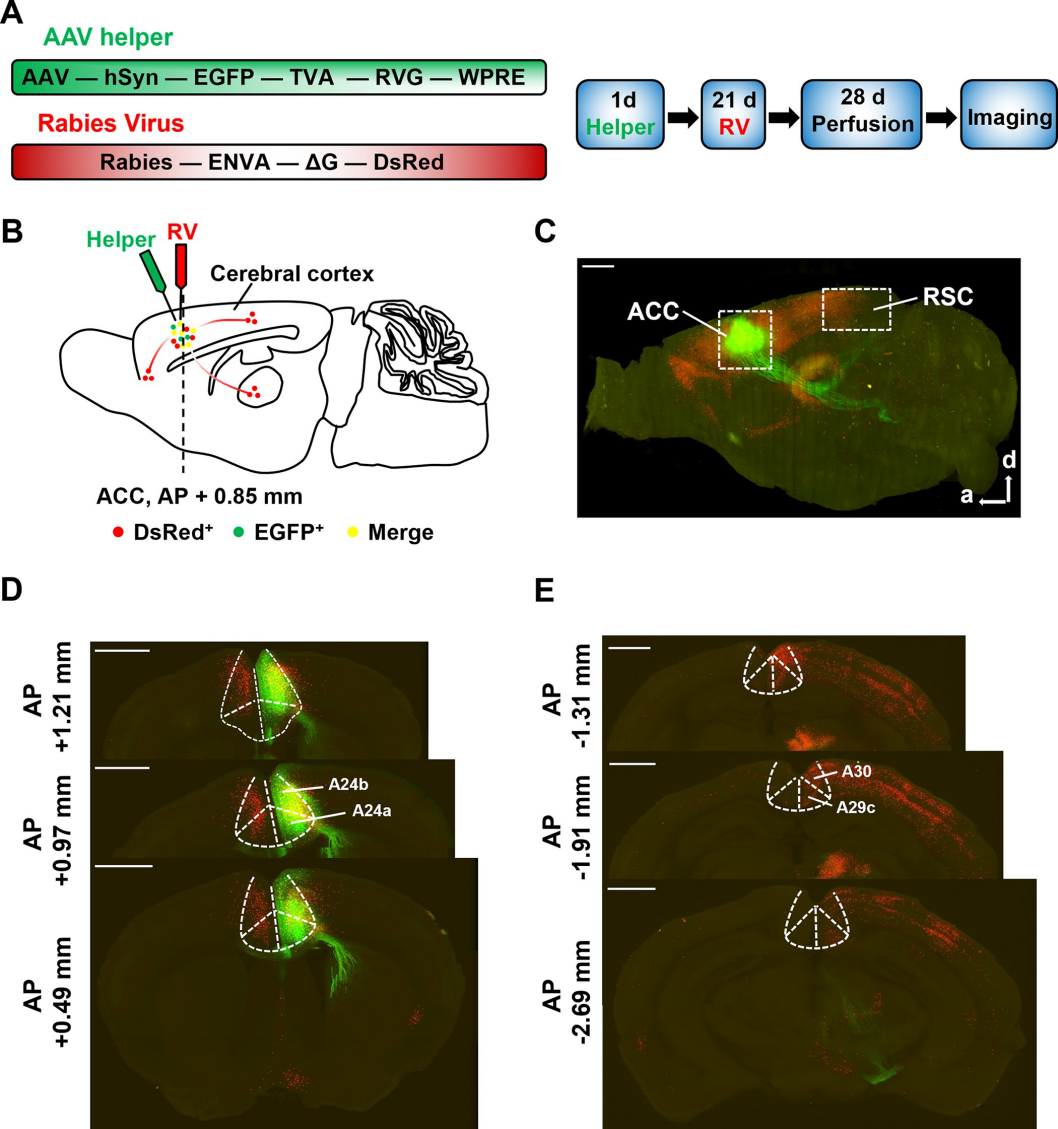
The ACC receives a substantial number of projections from the RSC. ** (A)** The trans-monosynaptic retrograde tracing virus and the experimental schedule used in the tracing strategy. **Left**: the AAV helper virus with TVA receptor, RVG, and EGFP. EnvA-pseudotyped glycoprotein (G)-deleted rabies virus with DsRed. **Right**: the timeline of the virus injection and the preparation of the brain slice for retrograde trans-monosynaptic tracing. **(B)** Schematic diagram of sagittal section for showing the injection site of the AAV helper virus (green) and rabies virus (red) separately in the unilateral ACC, and the labeling of direct afferents to the ACC in the whole brain. **(C)** The sagittal view of 3D-reconstructed whole-brain labeling in VISoR imaging (red: DsRed^+^; green: EGFP^+^). The white dashed boxes represent the regions of ACC and RSC in the brain. Scale bar: 1 mm. a: anterior; d: dorsal. **(D, E)** Representative coronal sections of VISoR imaging in the ACC (cortical areas 24a and 24b) **(D)** and RSC (A29c and A30) **(E)**. *n* = 3 mice. Scale bar: 1 mm. ACC, anterior cingulate cortex; RSC, retrosplenial cortex; VISoR, Volumetric Imaging with Synchronized on-the-fly-scan and Readout.

Recently, the large-scale 3D imaging technique has been quite mature and popular in the systematic brain mapping of different animals. The VISoR system achieves high-speed, high-resolution, high-throughput 3D whole-brain imaging for a better understanding of brain architecture and functions [29,30]. Here, we used VISoR imaging combined with a rabies virus-based trans-monosynaptic tracing strategy to label all the afferent projections to the ACC in adult mice. The AAV helper virus (rAAV-hSyn-EGFP-2a-TVA-2a-RVG-WPREs-pA) was first micro-injected into the unilateral ACC. After 3 weeks, the rabies virus (RV-EnvA-ΔG-DsRed) was also stereotaxically micro-injected into the same site, which retrogradely spreads to the upstream neurons in a trans-monosynaptic way with the assistance of AAV helper virus (Fig 1A and 1B). Seven days later, the mouse brain was separated and cut into 40 to 50 coronal slices with 300-μm thickness each for tissue clearing. These cleared coronal brain slices were imaged in sequence with the VISoR technique, and the whole-brain 3D reconstruction was performed after collecting all the volumetric imaging data.

In the sagittal view, the injection site in the ACC and the target regions were observed clearly and stereoscopically (Fig 1C). In the ACC, the starter neurons were both infected by the helper virus (EGFP^+^, green) and rabies virus (DsRed^+^, red) (Fig 1C and 1D). Target regions showing DsRed^+^ represented as the presynaptic inputs to the starter neurons of the ACC (Fig 1C–1E). In the coronal sections, we found that the unilateral ACC received the afferent projections from both the granular (A29c) and agranular (A30) areas of the bilateral RSC (Fig 1D and 1E). However, the projections from the ipsilateral RSC to ACC were significantly greater than those from the contralateral RSC. These results provide direct evidence for the spatial projections from the RSC to the ACC.

### Multichannel field potential recording reveals the excitatory connections from the RSC to the ACC

**Fig 2:**
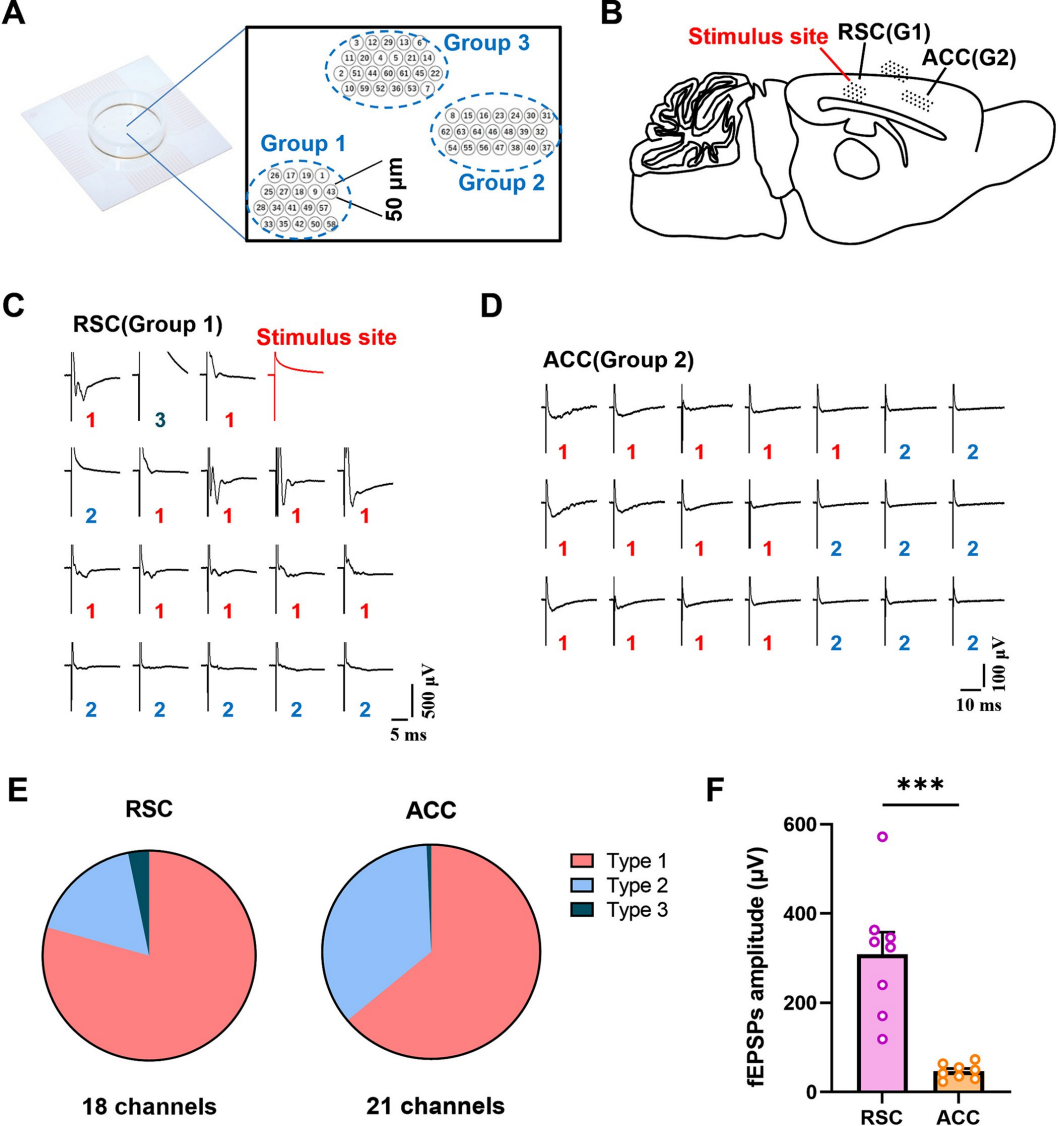
The long-distance RSC-ACC projections are dominantly excitatory in the multichannel field potential recordings. Schematic diagram of the MED64 P5001A probe (three-group arrays including 64 50 × 50-μm planar microelectrodes with 150 μm apart from each other). **(B)** Schematic diagram showing the relative position between the multichannel probe and the sagittal brain slice. The fEPSPs of RSC and ACC regions are recorded respectively by Group 1 (G1) and Group 2 (G2) electrode arrays with the stimulus site (red) in the RSC. **(C, D)** The example of recorded fEPSPs from the RSC (18 channels) and ACC (21 channels) with one stimulus channel (red line) in the RSC. The numbers 1–3 represent different types of channels (the active, the silent, and abnormal channels, respectively). **(E)** The pie chart showing the percentage of 3 types of the recorded channels in the RSC and ACC, respectively (RSC: type 1 = 79.37%, type 2 = 17.46%, type 3 = 3.17%; ACC: type 1 = 63.95%, type 2 = 35.37%, type 3 = 0.68%, *n =* 7 slices from 5 mice). **(F)** The averaged amplitude of all the recorded fEPSCs from the RSC and ACC in each brain slice (two-tailed unpaired *t* test: *t* = 5.314, ***p < 0.0001, *n =* 8 slices from 5 mice). The summary data for Fig 2 can be found in S1 Data. ACC, anterior cingulate cortex; RSC, retrosplenial cortex.

Although the imaging results verified the anatomic connections from the RSC to the ACC, the physiological features of these projections are still unknown. So far, most studies in the RSC-ACC pathway have mainly focused on anatomic and functional neuroimaging studies [31,32]. Few studies reported its related electrophysiological and synaptic mechanisms. Here, we first performed the multichannel field potential recording (MED64) to record the field excitatory postsynaptic potentials (fEPSPs) in the ACC with the electrical stimulus in the RSC on the sagittal brain slice (Fig 2A and 2B). Among 64 microelectrodes, 19 channels (Group 1) were placed in the RSC and 21 channels (Group 2) in the ACC. As shown in the example traces (Fig 2C), the red-line channel was chosen as the stimulus site and the other 18 channels (Group 1) were used for recording the evoked fEPSPs in the RSC. The fEPSPs of the RSC were classified into 3 types of responses according to their amplitude and shapes: 11 active channels (Type 1, red numbers), 6 silent channels (Type 2, blue numbers), and 1 abnormal channel (Type 3, dark cyan numbers) due to the microelectrode damage or poor hydrophilicity (Fig 2C). The evoked fEPSPs were also recorded in the ACC (Fig 2D). Among all 21 channels (Group 2), there were 13 active channels (Type 1) and 8 silent channels (Type 2). The percentage of 3 types of fEPSPs recorded from all the slices was summarized in Fig 2E. Obviously, the fEPSP amplitude of different channels decreased with their distance to the stimulus site extending (Fig 2F). These results primarily suggest that the ACC region widely receives excitatory neural inputs from the RSC.

### Activation of the RSC increases postsynaptic Ca^2+^ concentration in the ACC neurons

**Fig 3:**
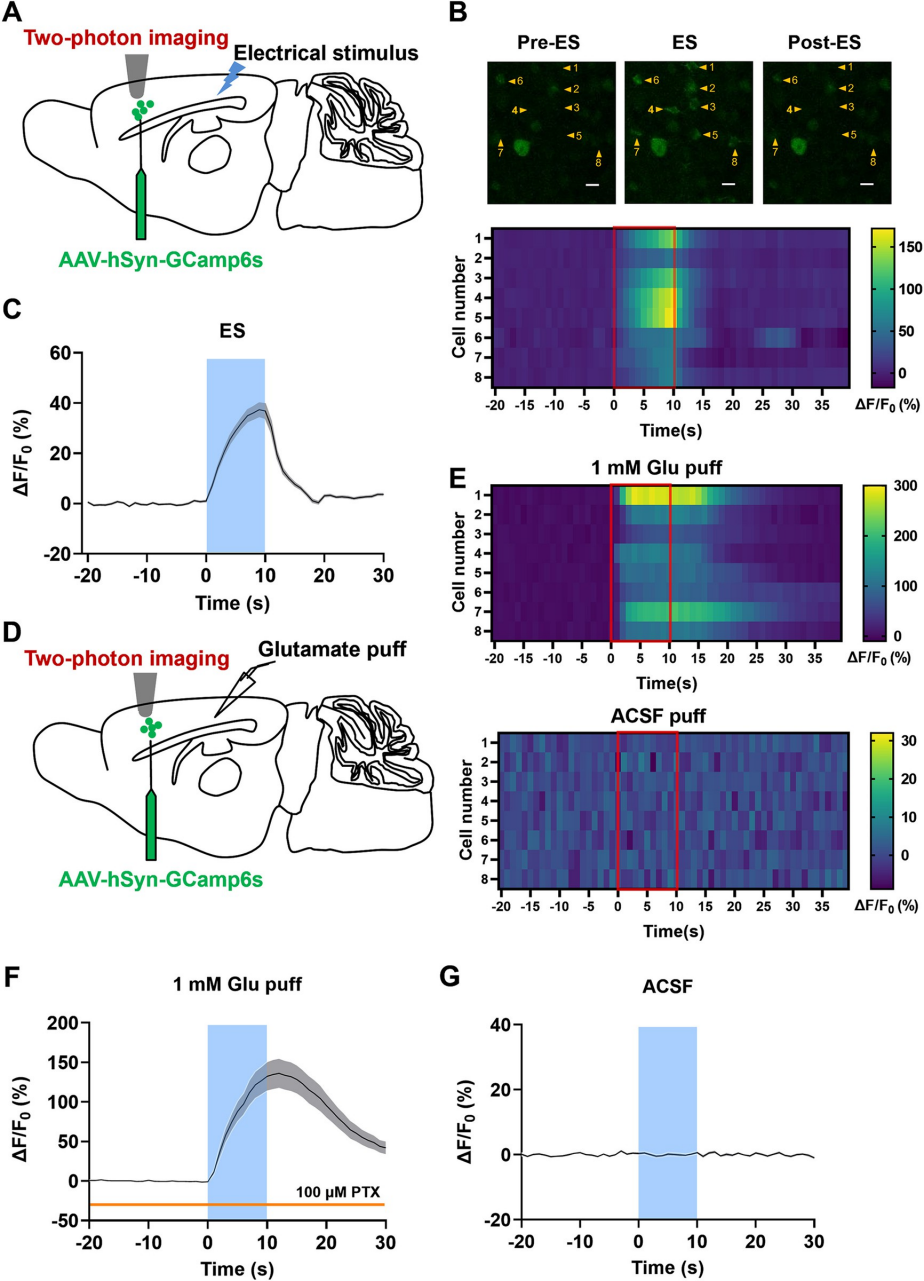
Activation of the RSC increases the intracellular Ca^2+^ concentration of the downstream ACC neurons. **(A)** Schematic diagram showing the recording site in the ACC and the placement of the stimulating electrode in the RSC. **(B)** The representative fluorescence changes (above) and the corresponding heatmap (below) of the somatic Ca^2+^ signals (ΔF/F_0_) in the ACC evoked by a 10 s electrical stimulus (ES) in the RSC. The numbers 1–8 represent different ACC neurons. The red rectangle area indicates the phase of the electrical stimulus. **(C)** Averaged fluorescence response of Ca^2+^ signal traces (ΔF/F_0_) from 141 ACC neurons of 3 mice with a 10 s electrical stimulus in the RSC. The blue area indicates the stimulus phase. The gray-shaded area indicates SEM. **(D)** Schematic diagram showing the recording site of two-photon Ca^2+^ imaging in the ACC and the site of local puff application of 1 mM glutamate in the RSC. **(E)** Representative heatmaps of fluorescence changes of Ca^2+^ signals from the ACC neurons with puff-applied glutamate (above) and ACSF (below) in the RSC. The red rectangle areas indicate the phase of puff application (10 s). **(F, G)** Averaged traces of Ca^2+^ signal change with glutamate **(F)** or ACSF **(G)** puff in the RSC (*n =* 52 cells from 3 mice). ACC, anterior cingulate cortex; RSC, retrosplenial cortex.

Intracellular calcium level is quite critical for synaptic transmission and plasticity, and activity-induced calcium signals in the ACC neurons have been reported in our previous study [33]. To explore the changes in the postsynaptic calcium level in the RSC-ACC pathway, we used two-photon calcium imaging to detect the calcium signals in ACC neurons with different stimuli in the RSC. The GCaMP6s virus was micro-injected into the ACC for 2-week expression and the sagittal slices of the mouse brain were prepared for two-photon calcium imaging. Then, a bipolar tungsten stimulating electrode was placed into the RSC and released a 5-Hz 10-s pulse stimulation (Fig 3A). As the example shown in Fig 3B, the intracellular Ca^2+^ signals of 8 labeled neurons in the ACC raised obviously during the pulse stimulation. Most of the fluorescent signals returned to the baseline levels soon after the stimulation (Fig 3B, below). In a total of 141 activated neurons, the peak of GCaMP6s signal intensity (ΔF/F_0_) at the end of stimulation was 37.30 ± 2.73% higher than the baseline (*n =* 4 slices from 3 mice) (Fig 3C).

To exclude the possibility of activating the passing fibers in the RSC by the electrical stimuli, we applied glutamate locally to activate the regional RSC by micro-injection (Fig 3D). We found that puff-application of glutamate (1 mM, 10 s, 5–10 psi) under the surface of the RSC region in the presence of picrotoxin (100 μm) also increased postsynaptic intracellular Ca^2+^ signals in the ACC. The heatmaps showed the example response that local glutamate puff in the RSC increased the intracellular Ca^2+^ signals in the ACC neurons, while the ACSF puff at the same site did not affect the fluorescent intensity (Fig 3E). The averaged response traces were shown in Fig 3F and 3G. The fluorescent intensity at the end of the glutamate puff was significantly higher than that of the ACSF puff (glutamate puff, 132.48 ± 18.56%; ACSF puff, 0.60 ± 0.47, two-tailed paired *t* test, *t* = 7.104, ***p < 0.001, *n =* 52 cells from 3 mice). These calcium imaging results indicate that activation of the RSC leads to the elevation of intracellular Ca^2+^ concentration in neurons of the downstream ACC.

### The electrophysiological characteristics of synaptic connection in the RSC-ACC projection

**Fig 4:**
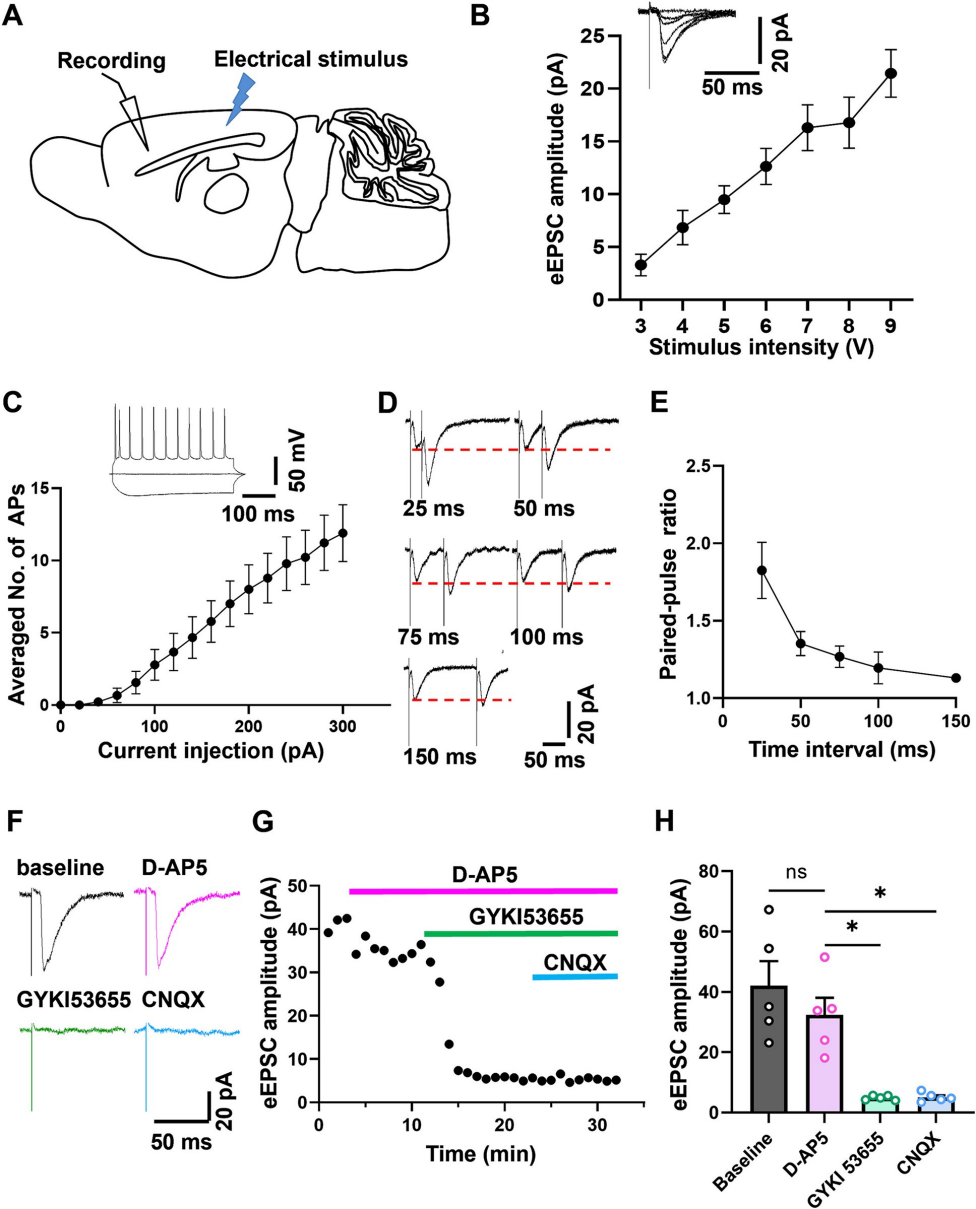
The excitatory transmission is mainly mediated by postsynaptic AMPA receptors in the RSC-ACC projections. **(A)** Schematic diagram showing the placement of stimulating and the recording electrode respectively in the RSC and ACC. **(B)** The input-output curve of the recorded pyramidal neurons with the holding membrane potential at −60 mV in the ACC. The inset showed the sample traces (*n* = 9 cells from 4 mice). **(C)** The averaged numbers of action potentials recorded in the pyramidal neurons with step current injections ranging from 0 pA to 300 pA in 20 pA increments and the sample traces above (*n* = 9 cells from 5 mice). **(D, E)** Representative traces **(D)** and the averaged curve **(E)** of PPRs with different intervals (25/50/75/100/150 ms) in the ACC neurons (*n* = 6 cells from 4 mice). **(F–H)** Sample traces **(F)**, sample amplitude curve **(G)**, and averaged amplitude **(H)** of eEPSCs in the RSC-ACC projections with the perfusion of D-AP5 (50 μm), GYKI53655 (100 μm), and CNQX (20 μm) sequentially under the presence of picrotoxin (50 μm) (one-way RM ANOVA with Sidak multiple comparisons tests, F = 20.44, *p* = 0.0027. Baseline vs. D-AP5, *p* = 0.6787; D-AP5 vs. GYKI 53655, *p = 0.0415 < 0.05; D-AP5 vs. CNQX, *p = 0.0346 < 0.05. *n* = 5 cells from 4 mice). ns means no significant difference. Error bars indicate SEM. The summary data for Fig 4 can be found in S2 Data. ACC, anterior cingulate cortex; eEPSC, evoked excitatory postsynaptic current; PPR, paired-pulse ratio; RSC, retrosplenial cortex.

To further study the characteristics of the RSC-ACC projection, the whole-cell patch-clamp recording was applied in ACC pyramidal neurons with a stimulus electrode in the RSC (Fig 4A). Local electric stimuli in the RSC induced intensity-dependent evoked excitatory postsynaptic currents (eEPSCs) in ACC pyramidal cells, which were shown in the inset of the input-output curve (I-O curve) (Fig 4B). The recorded action potentials (APs) under the current-clamp mode were used to confirm the ACC neurons as pyramidal neurons based on the firing pattern and shapes of their APs. The number of spikes had a positive correlation with the intensity of the step-depolarizing currents (Fig 4C). In addition, we also recorded the paired-pulse responses with different time intervals (25/50/75/100/150 ms). The paired-pulse ratio (PPR, the ratio of the amplitude of the second response to that of the first) decreased with the stimulus interval increasing (Fig 4D and 4E).

Previous studies in the ACC found that most EPSCs are mediated by AMPA and KA (kainate) receptors [34]. Next, we wanted to check if AMPA and KA receptors also contributed to synaptic transmission from the RSC to the ACC. The eEPSCs in the ACC were recorded in the presence of GABA_A_ receptor antagonist picrotoxin (100 μm). The selective NMDA receptor antagonist AP-5 (50 μm), selective AMPA receptor antagonist GYKI 53655 (100 μm), and AMPA/KA receptor antagonist CNQX (20 μm) were applied in sequence to detect the components of the EPSCs mediated by NMDA, AMPA, and KA receptor (Fig 4F and 4G). Only application of GYKI 53655 significantly decreased the amplitude of the eEPSCs, almost cutting off the excitatory transmission from the RSC to ACC. While the application of D-AP5 and CNQX had no effect on the responses (Fig 4H). These electrophysiological and pharmacological results reveal that ACC pyramidal neurons are innervated by the excitatory glutamatergic projections of the RSC, and this excitatory transmission is mainly mediated by postsynaptic AMPA receptors but not NMDA or KA receptors.

### Monosynaptic connections in the RSC-ACC projection

**Fig 5:**
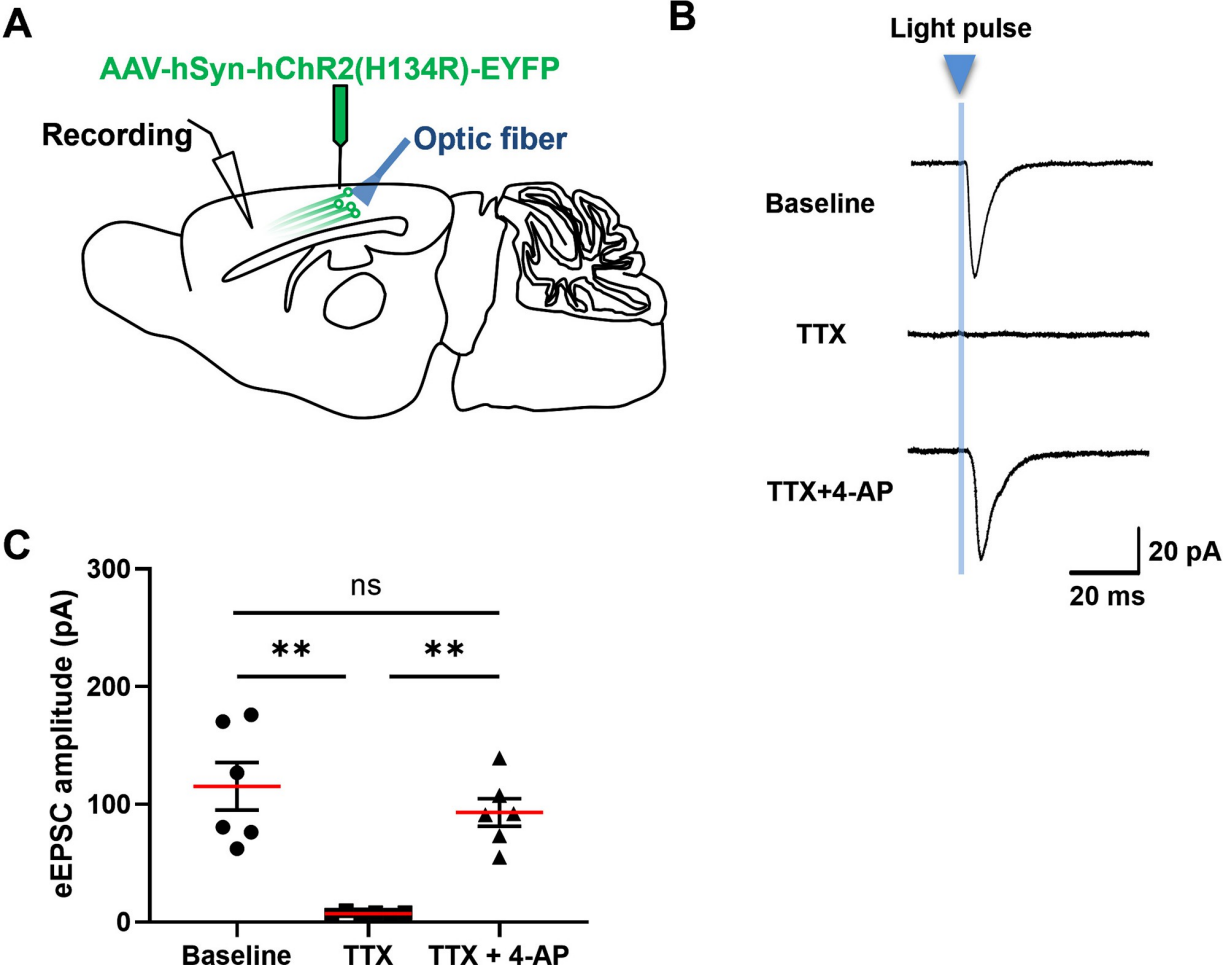
Monosynaptic connection from the RSC to ACC. **(A)** Schematic diagram showing the injection site of the optogenetic virus ChR2 and the patch-clamp recording site in the ACC. **(B)** The sample traces of eEPSCs induced by 0.5 ms blue-light pulse (the vertical blue line, 420~520 nm) with the perfusion of TTX (1 μm) and 4-AP (100 μm) sequentially. **(C)** The averaged amplitudes of eEPSCs at the baseline and after the application of TTX and 4-AP. 4-AP rescues the amplitude of eEPSCs almost totally blocked by TTX (one-way RM ANOVA with Sidak multiple comparisons tests, F = 18.17, *p* = 0.0023. Baseline vs. TTX, **p = 0.0088 < 0.01; TTX vs. TTX + 4-AP, **p = 0.0025 < 0.01. *n* = 6 cells from 3 mice). The red line indicates the mean value; ns means no significant difference. Error bars indicate SEM. The summary data for Fig 5 can be found in S3 Data. ACC, anterior cingulate cortex; eEPSC, evoked excitatory postsynaptic current; RSC, retrosplenial cortex.

To further identify whether the excitatory transmission from the RSC to the ACC is mono- or polysynaptic, we combined the electrophysiological technique with optogenetics to study this question. The optogenetic virus (AAV-hSyn-hChR2(H134R)-EYFP-WPREs-pA) was injected in the RSC for 2-week expression. The blue light-induced EPSCs were stably recorded in the ACC pyramidal neurons (Fig 5A). In the next pharmacological experiment, we first blocked voltage-gated Na^+^ channels by the perfusion of TTX (tetrodotoxin, 1 μm), then applied a nonselective K^+^ channel blocker 4-AP (100 μm) to block Kv channels in the brain slice. TTX can eliminate action potential-dependent EPSCs, blocking any polysynaptic transmission. Whereas the application of 4-AP enhanced the light-induced, direct depolarization of ChR2-positive nerve terminals, rescuing the eliminated eEPSCs (Fig 5B and 5C). These results confirmed the monosynaptic connections from the RSC to the ACC.

### The RSC-ACC projections modulate the mechanical and thermal nociception at a supraspinal level

**Fig 6:**
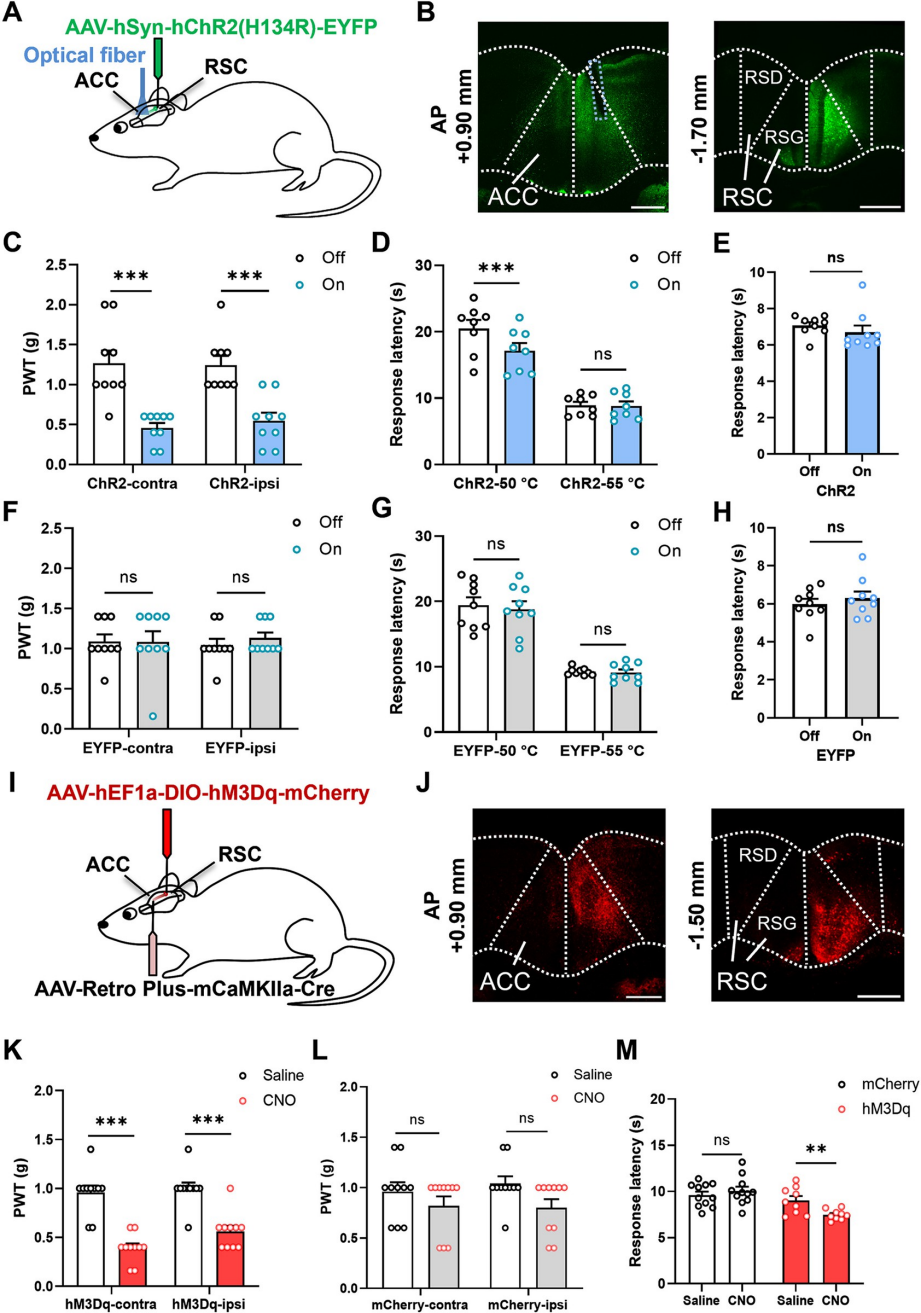
Activation of the RSC-ACC projections causes mechanical and thermal pain sensitization. **(A)** Schematic diagram showing the optogenetic paradigm for virus injection and optical fiber implantation in the brain. **(B)** Representative images showing the fluorescent expression of the optogenetic virus AAV-hSyn-hChR2(H134R)-EYFP in the ACC and RSC. The blue dashed line indicated the site of optical fiber implantation. Scale bar: 500 μm. **(C–H)** Bilateral mechanical PWT **(C and F)**, the response latency of the hindpaw lifting in 50°C and 55°C hot plate tests **(D and G)** and the response latency of the tail-flick tests **(E and H)** before and after blue-light stimulation. **(C and F)** Activation of RSC-ACC projections significantly decreases the bilateral PWT in ChR2 mice (two-way RM ANOVA with Sidak multiple comparisons test, light off vs. light on, F (1, 16) = 55.52, *p* < 0.0001, light off vs. light on in contralateral group, ***p < 0.0001, light off vs. light on in ipsilateral group, ***p = 0.0003 < 0.001, *n =* 9 mice), but not in the EYFP mice (two-way RM ANOVA with Sidak multiple comparisons test, light off vs. light on, F (1, 16) = 0.2682, *p* = 0.6116, light off vs. light on in contralateral group, *p* = 0.9991, light off vs. light on in ipsilateral group, *p* = 0.6997, *n =* 9 mice). **(D and G)** Activation of RSC-ACC projections decreases the response latency of 50°C hot plate test, but not 55°C hot plate test in the ChR2 mice (two-way RM ANOVA with Sidak multiple comparisons test, light off vs. light on, F (1, 14) = 15.46, *p* = 0.0015, light off vs. light on in 50°C group, ***p = 0.0002 < 0.001, light off vs. light on in 55°C group, *p* = 0.9807, *n =* 8 mice). Blue-light stimuli do not affect the behavioral performance of the EYFP mice in both 50 and 55°C hot plate tests (two-way RM ANOVA with Sidak multiple comparisons test, light off vs. light on, F (1, 16) = 0.5363, *p* = 0.4746, light off vs. light on in 50°C group, *p* = 0.6429, light off vs. light on in 55°C group, *p* = 0.9812, *n =* 9 mice). **(E and H)** Blue-light stimuli do not change the tail-flick response latency significantly in both ChR2 and EYFP mice (two-tailed paired *t* test, ChR2: *t* = 0.9925, *p* = 0.35, *n =* 9 mice; EYFP: *t* = 1.133, *p* = 0.29, *n =* 9 mice). **(I)** Schematic diagram showing the paradigm of the paired chemogenetic virus injection in the sagittal section of the mice brain. Two kinds of virus AAV-hEF1α-DIO-hM3Dq-mCherry and AAV-Retro Plus-mCaMKIIa-Cre are injected into the RSC and ACC respectively to specifically activate RSC-ACC excitatory neurons. **(J)** Representative images showing the fluorescent expression of the chemogenetic virus in the mice brain. Scale bar: 500 μm. **(K–M)** Bilateral PWT of the hM3Dq experimental and mCherry control mice **(K and L)**, the response latency of the hindpaw lifting in 55°C hot plate test **(M)** before and after applicating CNO (i.p., 2 mg/kg). **(K)** Specific activation of RSC-ACC excitatory neurons significantly decreases the bilateral PWT in the hM3Dq experimental mice (two-way RM ANOVA with Sidak multiple comparisons test, Saline vs. CNO, F (1, 18) = 83.4, *p* < 0.0001, Saline vs. CNO in contralateral group, ***p < 0.0001, Saline vs. CNO in ipsilateral group, ***p < 0.0001, *n =* 10 mice). **(L)** The application of CNO does not change the bilateral PWT obviously in the mCherry control mice (two-way RM ANOVA with Sidak multiple comparisons test, Saline vs. CNO, F (1, 18) = 3.919, *p* = 0.0632, Saline vs. CNO in contralateral group, *p* = 0.4459, Saline vs. CNO in ipsilateral group, *p* = 0.1077, *n =* 10 mice). **(M)** The response latency in the 55°C hot plate test was obviously reduced in the hM3Dq mice, but not changed in the mCherry mice (two-way RM ANOVA with Sidak multiple comparisons test, Saline vs. CNO, F (1, 18) = 4.148, *p* = 0.0567, Saline vs. CNO in mCherry group, *p* = 0.3943, *n =* 11 mice; Saline vs. CNO in hM3Dq group, **p = 0.0021 < 0.01, *n =* 9 mice). ns means no significant difference. Error bars indicate SEM. The summary data for Fig 6 can be found in S4 Data. ACC, anterior cingulate cortex; RSC, retrosplenial cortex.

Many studies show that ACC-related neuronal pathways are involved in pain processing and its related emotional disorders [2,3,17]. However, less is known about the detailed roles of RSC-ACC projections in pain modulation. Here, we perform optogenetic manipulations to find out whether the RSC-ACC neurons regulate pain-related behavioral responses. The optogenetic virus AAV-hSyn-hChR2(H134R)-EYFP was injected into the unilateral RSC with the optical fiber implanted into the ipsilateral ACC. Then, we can specifically activate the RSC-ACC projections by the blue-light (473 nm, 20 Hz) stimuli (Fig 6A). The projecting fibers and cell bodies of the virus-infected RSC neurons (EYFP^+^, green) were detected in the ACC and RSC, respectively (Fig 6B). The behavioral results of mechanical nociception showed that the mouse hindpaw withdrawal thresholds (PWT) in the light-on group were significantly decreased in bilateral hindpaws than that in the light-off group (Fig 6C). In the experiments of thermal nociception, the response latency of the hindpaw lifting in the 50°C hot plate test was also decreased with blue-light stimulation, but not changed in the 55°C hot plate test (Fig 6D). However, activating the RSC-ACC neurons did not affect the response latency of the acute thermal pain in the mouse tail-flick test, which usually represents a spinal nociceptive reflex without the regulation at a supraspinal level (Fig 6E). No matter whether with or without blue-light stimulation, all the mechanical and thermal nociceptive responses were not changed in the EYFP control mice (Fig 6F–6H).

To further investigate the roles of excitatory RSC-ACC projections in pain processing, 2 paired viruses (AAV-hEF1α-DIO-hM3Dq-mCherry and AAV-Retro Plus-mCaMKIIa-Cre) were injected into the RSC and ACC respectively to specifically activate the ipsilateral RSC-ACC excitatory neurons (Fig 6I). The expression of the chemogenetic viruses in the ACC and RSC were shown in Fig 6J. Similar to the optogenetic behavioral results, activating the unilateral RSC-ACC neurons by CNO (2 mg/kg, i.p.) reduced the bilateral mechanical withdrawal thresholds significantly in the hM3Dq mice (Fig 6K). However, the mechanical thresholds in the mCherry control mice (AAV-hEF1α-DIO-mCherry and AAV-Retro Plus-mCaMKIIa-Cre) were not reduced after the CNO application (Fig 6L). In the 55°C hot plate test, the CNO injection shortened the latency of tail-flick responses in the hM3Dq mice, but not in the mCherry control mice, confirming that the RSC-ACC excitatory projections involved in modulating thermal pain perception (Fig 6M). In summary, our behavioral results suggest that activation of the RSC-ACC excitatory projections leads to not only mechanical hyperalgesia but also thermal hypersensitivity in adult mice.

### Activating the RSC-ACC pathway has no effect on anxiety-like and aversive behaviors

**Fig 7:**
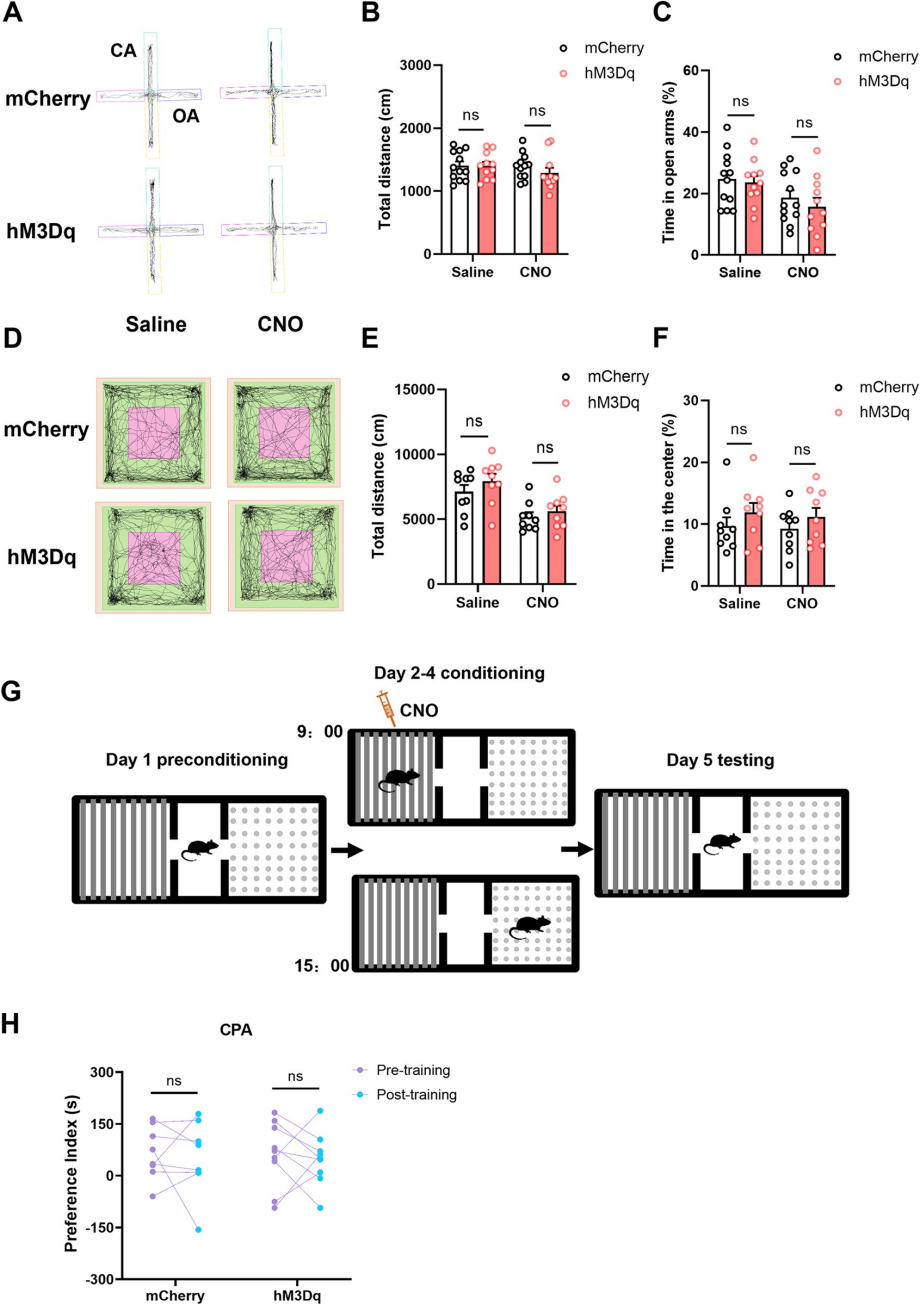
Activation of the RSC-ACC excitatory neurons does not affect anxiety-like and aversive behaviors in adult mice. (A) Representative traces showing the effect of CNO on movement of mice in the hM3Dq/mCherry mice in the EPM test. CA: close arm; OA: open arm. (B, C) The total movement distance and time in open arms of the mCherry and hM3Dq mice in the EPM test before and after CNO application. Activating the RSC-ACC excitatory neurons by CNO does not affect the movement of hM3Dq mice compared with the mCherry mice (total movement distance: two-way RM ANOVA with Sidak multiple comparisons test, mCherry vs. hM3Dq, F (1, 21) = 0.596, *p* = 0.449, mCherry vs. hM3Dq in Saline group, *p* = 0.999, *n =* 12 mice, mCherry vs. hM3Dq in CNO group, *p* = 0.46, *n =* 11 mice; Time in open arms: two-way RM ANOVA with Sidak multiple comparisons test, mCherry vs. hM3Dq, F (1, 21) = 0.5175, *p* = 0.4798, mCherry vs. hM3Dq in Saline group, *p* = 0.9394, *n =* 12 mice, mCherry vs. hM3Dq in CNO group, *p* = 0.6575, *n =* 11 mice). (D) Representative traces of 15 min movement of mCherry and hM3Dq mice in the open field test before and after CNO application. (E, F) The total movement distance and time in the center of the mCherry and hM3Dq mice in the open field test before and after CNO application. There is no difference in the movement between the mCherry and hM3Dq mice no matter before or after CNO application (Total movement distance: two-way RM ANOVA with Sidak multiple comparisons test, mCherry vs. hM3Dq, F (1, 16) = 1.25, *p* = 0.28, mCherry vs. hM3Dq in Saline group, *p* = 0.428, *n =* 9 mice, mCherry vs. hM3Dq in CNO group, *p* = 0.775, *n =* 9 mice; Time in the center: two-way RM ANOVA with Sidak multiple comparisons test, mCherry vs. hM3Dq, F (1, 16) = 1.40, *p* = 0.255, mCherry vs. hM3Dq in Saline group, *p* = 0.475, *n =* 9 mice, mCherry vs. hM3Dq in CNO group, *p* = 0.578, *n =* 9 mice). (G) The schematic diagram showing the paradigm of conditioned place aversion test in the mCherry and hM3Dq mice. The mice are put into one side of the shuttle box after CNO injection in every morning of the training day. (H) The preference index attained from the CPA test before and after CNO training in mCherry and hM3Dq groups. The 3-day application of CNO does not change the preference index of the mCherry and hM3Dq mice in the CPA test (two-way RM ANOVA with Sidak multiple comparisons test, Pre- vs. Post-training, F (1, 15) = 0.2987, *p* = 0.5928, Pre- vs. Post-training in mCherry group, *p* = 0.9122, *n =* 8 mice, Pre- vs. Post-training in hM3Dq group, *p* = 0.9132, *n =* 9 mice). ns means no significant difference, error bars indicate SEM. The summary data for Fig 7 can be found in S5 Data. ACC, anterior cingulate cortex; CPA, conditioned place aversion; EPM, elevated-plus maze; RSC, retrosplenial cortex.

Apart from nociceptive behaviors, we further examine whether the RSC-ACC pathway affects the pain-related negative emotions, such as anxiety or aversion, which are also modulated by the ACC [2,18]. The elevated-plus maze (EPM) and open field (OF) tests (Fig 7A–7F) combined with chemogenetics (Fig 6I) were utilized to assess the anxiety-like behaviors in the hM3Dq and mCherry mice. The representative traces of mouse movement in the EPM and OF test before and after CNO application were shown in Fig 7A and 7D. Activating the RSC-ACC excitatory neurons by chemogenetics did not change the movement and anxiety states (including the total travel distance and time in open arms) of the mCherry/hM3Dq mice in the EPM test (Fig 7B and 7C). Likewise, in the OF test, there was no difference in the total travel distance (Fig 7E) and time in the center (Fig 7F) before or after the CNO application. In the conditioned place aversion (CPA) test, the experimental paradigm is shown in Fig 7G; 3-day conditioning adaption on the same side of the box with CNO injection did not change the preference index in both mCherry and hM3Dq mice (Fig 7H). These results suggest that activating the RSC-ACC pathway is not necessary for the induction of anxiety and aversion in adult mice. The encoding of anxious and aversive feelings is probably separated from the pain processing of the RSC-ACC pathway.

### The RSC-ACC projections: A new cortico-cortical modulatory pathway for pain facilitation

**Fig 8:**
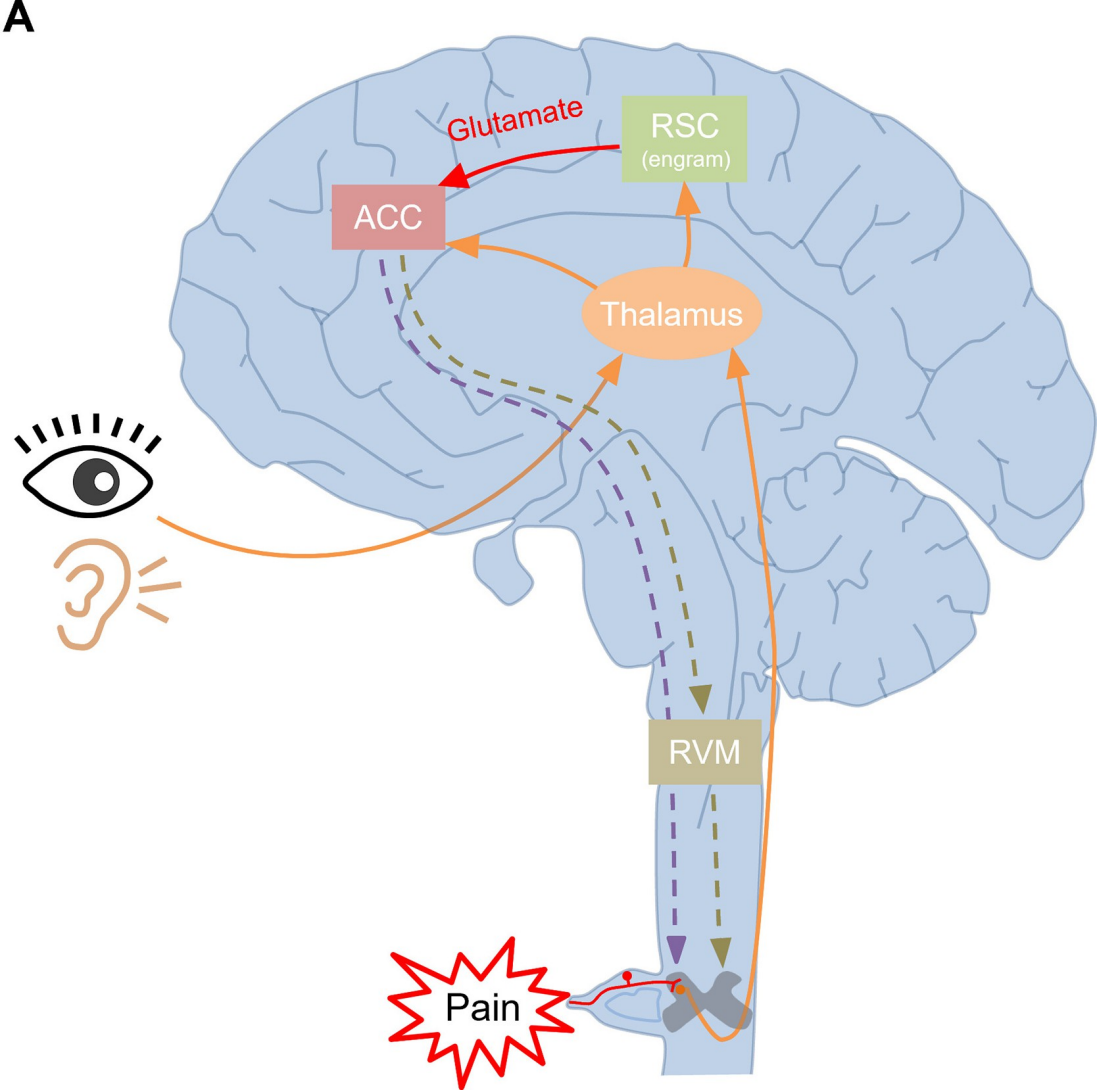
The new cortico-cortical modulatory pathway for pain facilitation. **(A)** A brief model for supraspinal facilitation of nociception by RSC-ACC excitatory pathway. The peripheral visual, auditory sensory, and somatosensory inputs (the orange lines) are conveyed to the thalamus. Then, the thalamus relays these sensory inputs to the cerebral cortex, such as the ACC and RSC. The ACC, as a high brain center for pain processing, is innervated by not only the subcortical thalamus but also the supracortical RSC (the red arrow lines) to integrate the nociceptive information. The excitatory RSC-ACC pathway probably involves in the combination of pain processing and memory (such as the pain-related contextual memory) by an engram mechanism in the RSC. The retrieved memory in the RSC can cause pain sensitization in the ACC, but not affect anxious or aversive feelings. Then, the high-activity ACC enhances the pain transmission and processing by recruiting distinct descending facilitatory pathways, such as the direct ACC-spinal cord or the indirect ACC-RVM-spinal cord pathway (the dash lines). ACC, anterior cingulate cortex; RSC, retrosplenial cortex; RVM, rostral ventromedial medulla.

As shown in Fig 8A, a brief model for supraspinal facilitation of nociception via RSC-ACC excitatory projections is proposed. The peripheral visual, auditory, and somatosensory inputs are conveyed to the thalamus and then relayed to the cerebral cortex, such as the ACC and the RSC. The RSC integrates these inputs and contributes to engram formation. Based on our results, the ACC, as a high brain center for pain processing, is also innervated by the excitatory glutamatergic projections from the RSC. These neuronal connections are monosynaptic and mediated mainly by presynaptic glutamate release and postsynaptic AMPA receptors. Meanwhile, the activation of the RSC-ACC pathway also elevates the intracellular Ca^2+^ concentration of postsynaptic pyramidal neurons in the ACC, affecting calcium-dependent synaptic plasticity. The excitatory RSC-ACC pathway probably links nociceptive perception to pain-related contextual memory by an engram mechanism in the RSC. In some similar scenes, the pain-related memory stored in the engram of the RSC gets retrieved and evokes mechanical and thermal pain sensitization, but not anxious or aversive feelings in the ACC. The hyperactivation of the ACC modulates peripheral pain transmission and processing by recruiting distinct descending facilitatory pathways, such as the direct ACC-spinal cord or the indirect ACC-RVM-spinal cord pathway, causing mechanical or thermal hyperalgesia. This physiological pain sensitization can raise our precautions and protect us from the coming dangers.

## Discussion

Accumulative evidence supports that the ACC is crucial for pain processing and pain-related emotional regulation [2,3,18]. The activity of excitatory neurons, synaptic transmission, and plasticity in the ACC are thought to be the underlying mechanisms of pain processing and its related emotional disorders [3]. In recent years, the exploration of interactions between the ACC and various other brain regions remains a central focus in the field of pain and pain-related research. However, there is still a lack of detailed electrophysiological and behavioral relevant data and understanding in the regulatory network of pain and pain-related emotions. In this study, we identified a new cortico-cortical pathway that contributes to acute mechanical and thermal nociceptive perception in mice. The ACC received the direct excitatory projections from the RSC. This excitatory synaptic transmission is mainly mediated by postsynaptic AMPA receptors and coupled with the elevation of the intracellular Ca^2+^ level in the ACC. Phenotypically, activating the RSC-ACC pathway facilitates both mechanical and thermal nociceptive perception at a supraspinal level without affecting spinal nociceptive transmission directly. Nevertheless, this pathway did not participate in the regulation of anxiety-like and aversive behaviors. It is the first report to reveal the specific nociceptive facilitation mediated by the RSC-ACC pathway. This finding suggests that the processing of pain and pain-related emotional information in the brain may be regioselective and separated among different cortical areas.

### The ACC-mediated network for pain and emotion regulation

As a hub for processing sensory, emotional, and cognitive information, ACC integrates plenty of neural inputs from various brain regions, including sensory, limbic, and cortical brain regions, etc., distinctly contributing to pain and pain-related emotions [3,35]. Recent research has demonstrated robust connections from the thalamus to the ACC in mice. Specifically, the ACC receives extensive projections from multiple thalamic subnuclei, including the anterior, ventral, medial, and lateral thalamic nuclei [36]. Electrophysiological investigations have revealed that stimulation of the thalamus induces short-term plastic changes in the ACC, facilitating the transmission of nociceptive information [4,37,38]. Moreover, the inputs from the mediodorsal thalamus (MD) to the ACC have been reported to elicit chronic pain-related aversion [39]. Besides the main inputs from the thalamus, the amygdala can relay the ascending nociceptive information to the ACC for pain and pain-related emotion regulation [3,39,40]. The pathway from the basolateral nucleus of the amygdala (BLA) to the ACC is crucial for the regulation of chronic pain and depression comorbidity [40]. Another study proved that activation of the ascending locus coeruleus(LC)-ACC noradrenergic projections facilitated pain and itch responses by enhancing glutamatergic synaptic transmission and neural excitability within the ACC [41]. Conversely, the GABAergic projections from the PVN and medial prefrontal cortex (mPFC) to ACC are found to attenuate neuropathic pain as well as comorbid anxiety behaviors by different types of interneurons [7,22]. In our research, we identified a novel cortico-cortical neural pathway that functions in the mediation of pain perception, contributing to a better understanding of the pain regulatory network within the brain. We observed that bilateral neurons in the ACC receive direct excitatory input from the unilateral RSC. Furthermore, optogenetic stimulation of the unilateral RSC-ACC projections resulted in a decrease in the bilateral mechanical thresholds of mouse hindpaws. According to another study, the ACC-ACC excitatory connections (from ipsilateral to contralateral side crossing the corpus callosum) contribute to bilateral pain perception. These findings may explain the reason why unilateral activation of the RSC-ACC pathway induces bilateral allodynia of mice in our behavioral tests.

### Synaptic characteristics in cortico-cortical connections

Although many anatomic studies reveal the projections from different cortical and subcortical areas to the ACC [31,32,36]. Few studies have provided direct evidence to describe the characteristics of synaptic transmission of these projections. In the present study, we utilize electrophysiological techniques and optogenetics to explore the features of the RSC-ACC projection in vitro. Through sagittal brain slice preparations, we successfully recorded eEPSCs via electrical stimulation and optogenetics within the RSC. Further investigations confirmed that these synaptic connections were monosynaptic. Pharmacological assays using different glutamate receptor antagonists demonstrated that the eEPSCs in the ACC are primarily mediated by AMPA receptors without any component of KA receptors. This finding contrasts with typical scenarios where the part of residual eEPSCs, induced by in situ, contralateral, or thalamic stimulation, were mediated by KA receptors [34,42]. Wu and colleagues identified these residual currents and established their mediation through GluK1 and GluK2 receptors in specific KA receptor knockout mice [34]. This indicates a heterogeneity of cortical synapses within the ACC. Distinct synaptic connections are responsible for distinct functions. We propose at least 3 different types of excitatory synapses: silent synapses; AMPA receptor-containing synapses; and mixed synapses comprising both AMPA and KA receptors. Although direct evidence for silent synapses in the ACC remains elusive [43], our previous electrophysiological data consistently suggested not only their existence or pure NMDA receptor-mediated responses but also their recruitment through long-term potentiation (LTP) or chemical induction [44–46]. Our findings provide new evidence supporting pure AMPA receptor-containing excitatory synapses, similar to well-characterized excitatory synapses found in the CA1 region of the hippocampus [47]. However, it is unclear if such AMPA synapses are common in other cortico-cortical connections. Lastly, we also observed that the projections from side-to-side ACC contain mixed-type synapses expressing both AMPA and KA receptors [48]. Comprehensive studies are warranted to delineate the characteristics of these diverse synapse types throughout the entire brain rather than focusing solely on the ACC.

### The modulation of intracellular Ca^2+^ signal in the ACC

It is well established that intracellular Ca^2+^ and Ca^2+^ channels play pivotal roles in neural excitability and function, particularly in neurotransmitter release and synaptic transmission [49,50]. In neurons, cytosolic Ca^2+^ influences various downstream signaling pathways and gene expression to regulate cellular physiology and development. For example, elevated cytosolic Ca^2+^ concentrations can activate classical PKA/PKC/CaMKII signaling cascades, leading to cAMP-response element binding protein (CREB) activation and promoting protein synthesis-dependent synaptic plasticity such as LTP [3,51–53]. Our previous study provided electrophysiological evidence indicating that N-type voltage-gated calcium channels (VGCCs) primarily mediate fast synaptic transmission within ACC [54]. Regarding postsynaptic Ca^2+^ dynamics, Li and colleagues reported that both NMDA receptors and VGCC-mediated action potentials induce significant Ca^2+^ influx in ACC pyramidal neurons. This influx was also observed during the induction phase of LTP, emphasizing the close relationship between intracellular Ca^2+^ signaling pathways and synaptic plasticity [33]. Utilizing in vivo Ca^2+^ imaging techniques on awake mice, a recent study demonstrated that increased amplitude of the Ca^2+^ signal in layer 5 pyramidal neurons of the ACC correlates with mechanical stimulation intensity. A single noxious pain stimulus elicited substantial Ca^2+^ transients; conversely, non-noxious stimuli had negligible effects in the ACC [55]. Collectively, these findings suggest that Ca^2+^ channels along with their associated signaling pathways significantly contribute to pain processing in ACC neurons. In this study, we discovered that activation of the RSC-ACC projections markedly elevates cytosolic Ca^2+^ levels in ACC pyramidal neurons—potentially influencing changes in synaptic plasticity such as LTP or silent synapse recruitment. We are continuing our investigation into how these calcium signaling pathways regulate the RSC-ACC connectivity.

### Functional implications of the RSC-ACC pathway

RSC has been implicated in various high-level cognitive functions, including spatial navigation, associative learning, and memory formation [25,26,56,57]. Numerous prior studies have indicated a close relationship between the RSC and nociceptive perception [27,28,58,59]. Electrophysiological recordings demonstrated enhanced responses of RSC neurons to cutaneous noxious stimulation in rabbits [58]. While most research involving both humans and animals suggests that the ACC and insular cortex (IC) play critical roles in pain perception and chronic pain management, it is inferred that the role of the RSC may be preferentially modulatory rather than central [6,9,60]. However, our findings provide direct and compelling evidence that excitatory projections from the RSC to ACC are also significant for nociceptive processing under physiological conditions. Activation of the RSC-ACC pathway facilitated behavioral responses to both noxious mechanical and thermal stimuli in naturally behaving mice. It has been reported that there are two descending pathways contributing to nociceptive facilitation: via direct cortical-spinal projections [61] or brainstem relay [62–64]. However, this conflicts with our behavioral results. According to our behavioral results, activation of the RSC-ACC pathway did not influence response latency in the spinal tail-flick test. We consider that the RSC-ACC connection modulates perceptions of both mechanical and thermal nociception at a supraspinal level. This modulation occurs through the direct excitation of ACC neurons without affecting descending facilitatory mechanisms to amplify the nociceptive inputs. Our results align with the theory that forebrain regions are essential for integrating sensory inputs with motor responses, which is necessary for withdrawal behaviors in the hot-plate test [65]. In our hot plate tests, we found that activating all the RSC-ACC projecting neurons by hSyn-ChR2 only facilitated the withdrawal responses to 50°C, but not to 55°C thermal stimuli. However, specifically activating the excitatory projecting neurons by CaMKII-hM3Dq significantly decreased the response latency in the 55°C hot plate test. Besides the methodological differences, we think that hSyn promoter may activate other potential regulatory components, such as interneurons or neuropeptides, probably leading to the differences of thermal sensitization which could be only detected in 50°C hot plate test. The roles of the other neural components in the RSC-ACC pathway are required to be further explored. Our work thus offers a more comprehensive understanding of regulatory networks involved in pain processes—particularly among different cortices. Consequently, we propose a novel mechanism whereby cognitive or non-sensory responses interact with nociceptive perception without disrupting spinal sensory transmission or reflexive actions. Additionally, it is posited that the RSC-ACC pathway is involved in modulating pain-related contextual memory due to its nociceptive facilitation under physiological conditions.

In summary, we have identified a novel direct nociceptive pathway connecting the RSC to the ACC. The activation of this pathway can facilitate supraspinal nociceptive processing. Notably, our findings confirm the heterogeneity of cortical glutamatergic synapses in the brain. This heterogeneity undoubtedly contributes to the complexity of cortical functions. Furthermore, our results elucidate a new mechanism for the selective enhancement of supraspinal pain processing, which occurs without influencing spinal reflexive responses.

### Experimental model and subject details

#### Animals

Adult (aged 6 to 8 weeks) C57BL/6 male mice were purchased from the Experimental Animal Center of Fujian Medical University. Experimental animals were housed in plastic cages with ad libitum access to enough water and mouse chow, and the holding room was kept under standard laboratory conditions (12 h/12 h day/night cycle, temperature of 22 to 25°C, air humidity of 55% to 60%). Mice were raised under standard laboratory conditions at least 1 week before all animal experiments were carried out. All experimental procedures were performed following the guidelines approved by the Ethics Committee of Fujian Medical University and Forevercheer Medicine Pharmac Inc. (Qingdao). The license number of the ethical approval for the animal experiments: IACUC FJMU 2024-Y^-0586^ and ECAU FMPI 2023020627.

#### Drug application

All the chemicals and drugs were obtained from Tocris Cookson (Bristol, United Kingdom) and MedChemExpress (New Jersey, United States of America). Selective competitive NMDA receptor antagonist D-AP5 was prepared in distilled water. Non-competitive AMPA receptor antagonist GYKI53655 hydrochloride and selective non-NMDA ionotropic glutamate receptors antagonist CNQX were dissolved in dimethyl sulfoxide (DMSO). GABA_A_ receptor antagonist Picrotoxin was dissolved in ethanol as a stock solution. 4-AP was dissolved in distilled water with ultrasonic assistance. All these stock solutions were diluted to the final desired concentration in the artificial cerebrospinal fluid (ACSF) before immediate use. The DMSO and ethanol diluted in ACSF did not affect basal synaptic transmission and plasticity.

#### Anatomy and imaging

In the trans-monosynaptic retrograde tracing experiments, the viruses AAV2/9-hSyn-EGFP-2a-TVA-2a-RVG-WPRE-pA (200 nL, 2.0 × 10^12^ genomics copies/ml, brainvta) and RV-EnvA-ΔG-DsRed (2.0 × 10^8^ genomic copies/ml, brainvta) were separately injected into the right ACC. After a 3-week expression, the mice with the full viral infection were deeply anesthetized and perfused with 0.01 M PBS, followed by 100 ml of 4% PFA in PBS (pH 7.4). The whole brain was immediately separated and stored in the 4% PFA solution for 4-h post-fixation. Then, the whole brain was placed into 0.1 M PB containing 30% (w/v) sucrose solution for 3-d dehydration at 4°C and cut into 30 μm-thickness coronal brain slices using a freezing microtome (Leica CM1900). Sections were collected in sequence and every third section was mounted onto the slides. These sections were counterstained with DAPI (ABS9235, absin, Shanghai) and observed using a laser scanning confocal microscope (FV3000, Olympus, Japan) or a slide scanner (Slideview VS200, Olympus).

For trans-monosynaptic retrograde tracing experiments, we used a fast and high-resolution VISoR imaging method as previously described [29,30]. The separated whole brain was placed into 4% acrylamide hydrogel monomer solution (w/v, HMS) in PBS for 2 days at 4°C. Next, the whole brain was embedded with equal volume mixed solution containing 4% HMS and 20% bovine serum albumin (BSA) at 37°C for 4 h and cut into 300-μm thickness coronal sections. These sections were transferred into 5% PBS-Triton clearing solution for 24 h at 37°C with gentle shaking to increase membrane permeability. After clearing, these sections were washed 3 times with PBS and mounted onto the quartz slides in sequence. The quartz slide with fixed sections was immersed into the refractive-index-matching solution and these sections were visualized with synchronized beam-scan illumination and camera-frame readout (10× objective). The resultant voxel size is 0.5 × 0.5 × 3.5 μm^3^.

#### Multichannel field potential recordings

For extracellular field potential recordings, we performed a 64-channel recording system (MED64, Alpha-Med Sciences, Japan) throughout the experiments as previously described [46]. The MED64 P5001A probe contained 64 planar microelectrodes (50 × 50 μm/each) with a 150-μm interpolar distance. Before experiments, the surface of the MED64 P5001A probe was pre-treated overnight with 0.1% polyethyleneimine (Sigma Aldrich, St. Louis, MO; P^-3143^) in 25 mM borate buffer (pH 8.4) at room temperature to enhance surface hydrophilicity. The sagittal brain slice was prepared as mentioned above and transferred into the recording chamber after 1-h incubation. The ACC and RSC regions were covered separately onto the microelectrodes of P5001A probe and a fine mesh anchor was used to ensure slice stability during entire recordings. The slice was continuously perfused with oxygenated, fresh ACSF at 28 to 30°C and maintained at the flow rate of 2 to 3 ml/min throughout the entire experimental period. After a minimum 1-h recovery period, one channel located in the RSC was chosen as the optimum stimulus site, which can induce the best synaptic response in the ACC after a biphasic constant current pulse test stimulus (0.2 ms) was delivered. The channel with fEPSP induced by electrical stimulus was regarded as an activated channel. The fEPSP response was sampled once every minute and averaged every 2 traces.

#### Two-photon Ca^2+^ imaging

For two-photon calcium imaging, the virus AAV2/9-hSyn-GCamp6s-WPRE-hGHpA (150 nL, 1.6 × 10^12^ genomics copies/ml, brainvta) was injected into the bilateral ACC and expressed for at least 7 days. Two-photon Ca^2+^ imaging was performed by using a Scientifica Hyperscope with a 16 × 0.8 NA water-immersion lens (CFI75 LWD, Nikon) and Coherent laser (Chameleon Ultra II, tuning range from 680 to 1,080 nm, averaged power > 3.5 W) tuned at 900 nm for two-photon excitation for GCaMP6s. During two-photon imaging, the fluorescent baseline was first recorded at least for 20 s with the scanning parameters (1 frame/s and 512 × 512 pixels). After the baseline recording, different stimuli were applied in the RSC, at least 1.2 mm away from the imaging region in the ACC. The electrical stimulations (5 Hz, 5 ms pulse, and 195 ms interval, 9 V, 10 s) were delivered by a bipolar tungsten stimulating electrode in the RSC. In the puff experiments, 1 mM glutamate was released for 10 s just above the adjacent RSC through the whole-cell recording pipettes by using the MPPI-3 pressure injector (5~10 psi). The brain slices were perfused until the fluorescence was restored to the basal level after stimulus treatments. The obtained image data was analyzed with Image J. The fluorescent signals were quantified by measuring the mean pixel intensities of the cell body of each neuron. The fluorescent change was defined as ΔF/F_0_ = (F_t_−F_0_)/F_0_. F_t_ was the fluorescent intensity at time t, and F_0_ was the mean of the baseline intensity before the beginning of stimulus application.

#### In vitro whole-cell patch-clamp recordings

Briefly, mice were anesthetized with 2% isoflurane and decapitated quickly. The whole brain was rapidly separated and transferred into ice-cold oxygenated (95% O_2_ and 5% CO_2_) cutting solution (in mM: 252 sucrose, 2.5 KCl, 6 MgSO_4_, 0.5 CaCl_2_, 25 NaHCO_3_, 1.2 NaH_2_PO_4_, and 10 glucose, pH 7.3 to 7.4) within a short time. The whole brain was then trimmed and glued onto the ice-cold platform of a vibrating tissue slicer (Leica VT1200S). Then, 200-μm thickness sagittal brain slices containing both the RSC and ACC regions were cut (about 4 to 5 slices) according to the Mouse Brain in Stereotaxic Coordinates (4th edition) and then transferred to a room temperature-submerged incubation chamber containing oxygenated ACSF (in mM: 124 NaCl, 2.5 KCl, 1 NaH_2_PO_4_, 1 MgSO_4_, 2 CaCl_2_, 25 NaHCO_3_, and 10 glucose, pH 7.3 to 7.4) for at least 1-h incubation before conducting experiments.

The whole-cell patch recordings were performed as previously described [22]. The recordings were performed in voltage- or current-clamp mode using a HEKA amplifier. PatchMaster and Clampfit 10.2 software were used to acquire and analyze the data. In the present study, the eEPSCs were recorded in the ACC with a HEKA amplifier, and the electrical stimulations were delivered by a bipolar tungsten stimulating electrode placed in the RSC regions. For AMPA receptor-EPSCs and action potential recordings, the recording pipettes (3 to 5 MΩ for pyramidal neurons) were filled with an internal solution containing 124 mM K-gluconate, 5 mM NaCl, 1 mM MgCl_2_, 0.2 mM EGTA, 2 mM MgATP, 0.1 mM Na_3_GTP, and 10 mM HEPES (adjusted to pH 7.2 with KOH, 290 mOsmol). Picrotoxin (100 μm) was added to block GABA_A_ receptor-mediated inhibitory synaptic currents for EPSCs recordings in all experiments. The neurons were voltage clamped at −60 mV in the presence of D-AP5 (50 μm) for AMPA receptor-EPSCs recordings and both D-AP5 and GYKI53655 (100 μm) for KA receptor-EPSCs recordings. CNQX was added in ACSF to block selective non-NMDA ionotropic glutamate receptors. To examine synaptic responses, the I-O curves in the ACC pyramidal neurons were recorded at different stimulus intensities. To examine presynaptic functions, the PPRs were tested at different time intervals (25, 50, 75, 100, and 150 ms intervals). Action potentials were recorded in current-clamp mode by delivering stepped currents of −200 to 300 pA (400 ms duration) in increments of 20 pA. To verify the monosynaptic connections, TTX and 4-AP were added to unselectively block Na^+^ and K^+^ channels respectively for presynaptic depolarization which specifically promotes the monosynaptic glutamate release in the optogenetical experiment in vitro.

#### Virus injection and surgery

Virus injection procedures were performed as previously described [36]. The experimental mice were anesthetized with 2% isoflurane and fixed on a stereotaxic frame to keep parallel to the reference panel. A midline incision was made in the skull and the skull was drilled on the RSC (1.70 mm posterior to the bregma, 0.20 mm lateral to the midline, 1.00 mm ventral to the skull surface) or the ACC (0.90 mm anterior to the bregma, 0.30 mm lateral to the midline, 1.40 mm ventral to the skull surface). The viruses were stereotactically pressure-injected into the target site with equal speed (40 nL/min) using a microsyringe pump (Nanoject III #3-000-207, DRUMMOND). Next, a 10-min extension was allowed for diffusion of viral particles before the microsyringe was slowly withdrawn. The experimental mice were allowed to recover for at least 2 to 3 weeks before all the experiments were performed, except that the virus RV-EnvA-ΔG-DsRed was expressed for 7 days.

#### Optogenetic manipulations

For the in vitro electrophysiological experiment, the virus AAV2/9-hSyn-hChR2(H134R)-EYFP-WPRE-hGHpA (150 nL, 1.2 × 10^12^ genomics copies/ml, brainvta, Wuhan) was injected into the bilateral RSC and expressed for at least 2 weeks. The blue-light pulses (420~520 nm, 10~20 mW, 0.5 ms) were given by the pE-300 LED illumination system (CoolLED) for photostimulation. For the behavioral test, the virus AAV2/9-hSyn-hChR2(H134R)-EYFP-WPRE-hGHpA and AAV2/9-hSyn-EYFP-WPRE-hGHpA (150 nL, 1.2 × 10^12^ genomics copies/ml) was injected into the right RSC. The optic fiber cannula (length, 2 mm; 200-μm core; NA = 0.37; THINKERTECH, Nanjing) was chronically implanted into the ipsilateral ACC (0.90 mm anterior to the bregma, 0.40 mm lateral to the midline, 1.40 mm ventral to the skull surface) to activate neuronal terminals. Behavioral tests related to optogenetics were performed after a 2-week recovery. The optic fiber was connected to a fiber patch cable with a rotary joint, which was in turn connected to a fiber-coupled laser (200 mW, 465 nm, Inper Studio, Hangzhou). Mice received 465-nm blue-light illumination (5 to 20 mW, 20 Hz, 5 ms pulse) for the light-on group throughout the entire experiments in the EPM, OF tests. For von-Frey, tail-flick, and hot-plate tests, mice received blue-light illumination for 30 to 60 s before testing. Finally, all mice were sacrificed and the whole brain was sectioned to verify optic fiber implantation and viral expression. The data was excluded if the viral expression or optic fiber implantation had a deviation from the targeted regions.

#### Chemogenetic manipulations

For chemogenetic experiments, virus injection surgery was performed 2 weeks before the behavioral tests. The viruses AAV2/9-hEF1α-DIO-hM3Dq-mCherry-ER2-WPRE-pA / AAV2/9-hEF1α-DIO-mCherry-ER2-WPRE-pA and AAV2/2-Retro Plus-mCaMKIIa-Cre-WPRE-pA (120 nL, 2.0 × 10^12^ genomic copies/ml, Taitool, Shanghai) were injected into the right RSC and ACC respectively to label the excitatory neurons in the RSC-ACC pathway. CNO (MedChemExpress, 2 mg/kg, 0.1 ml/20 g body weight) or vehicle (saline) was injected intraperitoneally at least 30 min before behavioral testing. All the behavioral experiments were finished in 2 h after the CNO or vehicle injection. CNO was dissolved in the saline with ultrasonic assistance.

#### Mechanical withdrawal measurement

The mechanical hypersensitivity was determined using an up-down method with von Frey filaments (Stoelting; Wood Dale, Illinois) applied perpendicularly to the plantar surface as previously reported [22]. Mice were individually placed into a plastic cage with wire mesh floors and allowed to acclimate for 30 min before testing. A series of filaments (0.008, 0.02, 0.04, 0.16, 0.4, 0.6, 1, 1.4, 2.0 g) with various bending forces were applied to the plantar surface of the hindpaw until it was bent slightly and held for 3 s. Licking, biting, or sudden withdrawal of the hindpaw were defined as positive responses. An initial filament force of 0.4 g was applied to test if the mouse was sensitive to this force. If the positive response occurred, the filament force was incrementally decreased until a negative result was obtained with an interval of 3 to 5 min between 2 tests. If the mouse was insensitive to 0.4 g filament force, a stronger filament force was applied until a positive response was obtained. The hindpaw withdrawal thresholds were finally determined using the up-down method until the positive/negative responses crossed 5 times.

#### Hot plate test

The mouse was placed on a hot plate set at 50 ± 1°C or 55 ± 1°C. The latency time was recorded when the reaction of the hind paw (licking, shaking, or lifting) first appeared. The cut-off times (40 s for 50°C and 20 s for 55°C) were used to avoid tissue damage. Mice were tested a total of 3 times with an inter-trial interval of 10 min. The average of 3 repeated measurements was calculated as the final latency time.

#### Tail-flick test

The tail-flick reflex was measured using a 50 W projector lamp which produced noxious radiant heat. The TF latencies to reflexive removal of the tail from the heat were recorded for 3 repeated measurements with an inter-trial interval of 30 min. The cut-off time of 10 s was used to avoid heat damage to the tail.

#### Open field test

The open field test was performed as previously described [22]. The open field consisted of an opaque cube (40 × 40 × 30.5 cm) and was divided into a center zone (20 × 20 cm) and an outer zone as the periphery. A single mouse was placed into the arena center and allowed to explore freely for 15 min with dim illumination. The movement traces were tracked using tracking master v3.0 system and all measurements (total distance, time in the center, entries) were quantified relative to the mouse body.

#### Elevated plus maze

The EPM apparatus consisted of 2 open arms (30 × 5 cm) and 2 closed arms (30 × 5 × 30 cm) which were perpendicular to each other and intersected by a central platform (5 × 5 cm). The maze was 70 cm high from the floor. For each test, the mouse was individually placed into the center of the apparatus and allowed to explore freely for 5 min with dim illumination. A tracking master v3.0 system was used to track the mouse movement. The number of entries, time spent in the open arm, and total distance were quantified relative to the mouse body.

#### Conditioned place aversion test

In the conditioned place aversion test (CPA), the mice were preconditioned on the first days, and they were allowed to explore the chamber for 15 min freely. The time they spent in each chamber was recorded and analyzed. On the second day, the mice with the CNO injection were paired with a randomly chosen chamber for 15 min in the morning; 4 to 6 h later, the mice with the saline treatment were paired with the other chamber for 15 min in the afternoon. After 3 days of pairing, the mice were allowed to explore all the chambers for 15 min on the fifth day. The time spent in every chamber was analyzed to understand chamber preference. The preference index was calculated as the time spent in the CNO-paired chamber minus the time spent in the saline-paired chamber.

#### Statistical analysis

All data was reported as means ± SEM. OriginPro 2021, GraphPad Prism 9, and SPSS 22.0 software were used for figure plotting and data analysis. Two-tail paired or unpaired *t* test was used to examine statistical differences between the 2 groups. One-way ANOVA followed by Dunnett T3 post hoc test was used for comparison among multiple groups. Two-way ANOVA followed by Sidak multiple comparisons test was used to identify significant differences among multiple groups with 2 impact factors. The significance levels of the statistical tests were presented as *p < 0.05, **p < 0.01, ***p < 0.001; ns means no significance with *p* > 0.05.

## Supporting information

S1 Data Source data for Fig 2. All data points for Fig 2F.
(XLSX)

S2 Data Source data for Fig 4. All data points for Fig 4B, 4C, 4E, 4G, 4H.
(XLSX)

S3 Data Source data for Fig 5. All data points for Fig 5C.
(XLSX)

S4 Data Source data for Fig 6. All data points for Fig 6C–6H, 6K–6M.
(XLSX)

S5 Data Source data for Fig 7. All data points for Fig 7B, 7C, 7E, 7F, 7H.
(XLSX)

## Abbreviations

ACC: anterior cingulate cortex
ACSF: artificial cerebrospinal fluid
AP: action potential
BSA: bovine serum albumin
CPA: conditioned place aversion
eEPSC: evoked excitatory postsynaptic current
EPM: elevated-plus maze
HMS: hydrogel monomer solution
IC: insular cortex
LTP: long-term potentiation
MD: mediodorsal thalamus
mPFC: medial prefrontal cortex
NAc: nucleus accumbens
OF: open field
PPR: paired-pulse ratio
PVN: paraventricular nucleus
RSC: retrosplenial cortex
VGCC: voltage-gated calcium channel
VISoR: Volumetric Imaging with Synchronized on-the-fly-scan and Readout
VTA: ventral tegmental area
